# Population Mental Health and COVID-19: Why Do We Know So Little?

**DOI:** 10.1177/07067437211010523

**Published:** 2021-04-19

**Authors:** Scott B. Patten, Stanley Kutcher, David Streiner, David Gratzer, Paul Kurdyak, Lakshmi Yatham

**Affiliations:** 1 Department of Community Health Sciences, University of Calgary, Alberta, Canada; 2 Department of Psychiatry, Faculty of Medicine, Dalhousie University, Halifax, Nova Scotia, Canada; 3 Department of Psychiatry & Behavioural Neurosciences, McMaster University, Hamilton, Ontario, Canada; 4 Centre for Addiction and Mental Health, Toronto, Ontario, Canada; 5 The University of British Columbia, Vancouver, British Columbia, Canada

**Keywords:** epidemiology, general population, healthcare policy, health services, research, prevalence

Pandemics are believed to adversely affect mental health. Historically, first time hospitalization rates for mental disorders linked to influenza may have increased by a factor of 2.6 in the 5 years following Russian Influenza and by a factor of 7.2 yearly during the 6 years after the Spanish Flu.^1^ Similarly, the COVID-19 pandemic may create a “parallel pandemic” potentially including disorders such as posttraumatic stress disorder (PTSD) that may follow intensive care unit admissions, disorders related to widespread social effects, and neuropsychiatric effects of the virus itself. Yet, more than 1 year into the COVID-19 pandemic, the robust knowledge needed to inform policy and service delivery for mental health and mental disorders in Canada is not yet available. The purpose of this editorial is to provide a cautionary message about existing estimates, and to suggest strategies for generating better data during this pandemic, and beyond.

While Canada has a wealth of health administrative data that can be used to guide and evaluate various aspects of mental health services, there is currently little capacity to extract timely and salient information, especially at the national level. Apart from the logistical challenges involved, there is the additional problem that the pandemic has disrupted health systems in ways that complicate interpretation of administrative data. Healthcare and patient adaptations to address the COVID-19 crisis (such as the transition to virtual care) may account for changes in utilization in unpredictable directions. For example, there is evidence that a reduction in referrals early in the pandemic may be followed by acceleration in the medium term.^2^ There is thus a need to develop and expand infrastructure for collecting and managing health information and providing sophisticated and timely information. This must include the ability to disaggregate data to identify different segments of the population, such as racialized, marginalized, and indigenous populations. Indeed, this may be one of the most necessary learnings from this pandemic experience.^3^ A US study reported that those with COVID-19 had increased incidence (HR ranged from 1.6 to 2.1) of first psychiatric diagnoses during the 14 to 90 days after the COVID-19 diagnosis compared with 6 other health conditions,^4^ highlighting the utility of access to timely data.

Psychiatrists know that need for mental health care is a complex issue. Need is incompletely expressed by estimates of disorder prevalence or healthcare utilization. Need is also related to comorbidities, coping skills, personal resources, and so on. Mental disorders exist on a spectrum of severity. Given this context, information about the prevalence of mental disorders at best provides a starting point or bare minimum. Yet, no estimates of the prevalence of any mental disorders during the pandemic have appeared in Canada. Indeed, this lack of data is a global issue. Only a single study, conducted in the Czech Republic, used a structured diagnostic interview (the MINI) in repeated cross-sectional surveys (2017 and 2020) to assess prevalence before and during the pandemic. Increases of 2 to 3 fold in the point prevalence of anxiety disorders and depressive disorders, respectively, were reported.^5^

A commonly measured parameter has been perceived mental health, measured using a single item. About 1 in 4 Canadians reported fair or poor mental health during the pandemic, compared to about 8% in the 2018 Canadian Community Health Survey (CCHS). Whether and how such perceptions are related to need for mental health care is difficult to say.

Brief mental health screening scales having been commonly employed in the COVID-19 literature, especially the Patient Health Questionnaire (PHQ-9), the Generalized Anxiety Disorder Scale (GAD-7) and various versions of PTSD Checklist (PCL-5), for symptoms of depression, generalized anxiety and PTSD, respectively. Such instruments are inexpensive and convenient to administer. The problem is that they were designed for screening. Consequently, they are intended to be sensitive (so that cases are not missed) but are not intended to be specific since a positive screen leads to further assessment, not a diagnosis. By design, they produce a high proportion of false-positive ratings and tend to overestimate prevalence. Predictably, systematic reviews of studies that assessed prevalence of anxiety and depression using such scales have reported high prevalence estimates, as high as 31.9% for anxiety and 33.7% for depression.^6,7^ In Canada, the Perspectives series of surveys by Statistics Canada included the GAD-7, but there are no comparable pre-pandemic population values against which to interpret the scores. Population-based studies from the United States and UK have reported elevated non-specific distress ratings in general population samples assessed before and during the pandemic.^8^

An analysis of data collected from 3 cohorts 2 to 8 weeks into a COVID-19 lockdown in the Netherlands examined symptom changes from self-report ratings obtained before the pandemic in cohort members with and without prior diagnoses of mental disorders. While those with mental disorders had higher levels of symptoms during the lockdown, the largest increases in symptoms were observed in the groups without prior diagnoses.^9^ Whether these increased symptoms reflect de novo occurrence of disorders or stress-related changes in is unknown.

Mental disorders, as currently defined, are often associated with dysfunction of a normally adaptive process. Anxiety disorders are case in point—symptoms of anxiety facilitate preparation for threats and avoidance of danger. Self-reported anxiety or brief anxiety rating scales do not make this distinction. Anxiety symptoms measured by self-report during a pandemic may partially represent a normal and adaptive response to increased danger or stress and may be a less accurate marker of pathological states of anxiety.

Another set of uncertainties relate to sampling. The ability to make statistical inference is maximized by probability sampling. Each member of a random sample should have a known probability of selection if inferences about population characteristics are to be made. If the probability of selection is unknown, and especially if it may be influenced by characteristics under study, estimates may be biased. In view of this, it is disappointing that the vast majority of COVID-19 surveys have used convenience samples. Some or all of their published results may be wrong. Policy decisions based on these data may do harm rather than good and may create opportunity costs.

An additional methodological consideration is that of generalizability. Do the results of a study in one population have implications for other populations? Do estimates from jurisdictions with low case-numbers but strong mitigation strategies apply in the same way to those with higher case numbers and/or less strong mitigation strategies? Such questions are both interesting and important. However, the generalizability of invalid estimates (e.g., due to poor sampling procedures and/or weak measurement strategies) is a moot point since it would never be sensible to generalize an incorrect estimate to another population.

It is interesting to speculate why a medical-psychiatric establishment that has increasingly emphasized evidence-based approaches should seemingly abandon those principles at a time of crisis. A likely explanation is that there has been a perceived need to answer questions quickly. Special funding competitions, routine use of pre-print platforms, special issues of journals, and an openness to expedited publication have been evident. Such measures may make sense in the context of a public health emergency. However, it creates a risk that low-quality evidence is produced and acted upon as if it were high-quality evidence. This is problematic, especially if such studies appear in high-impact journals and are highly cited. Many surveys using convenience sampling and abbreviated measurement strategies have appeared in top-tier journals during the pandemic and have subsequently made headlines in media outlets. If it is reasonable that the rules of peer-review and parsing of evidence must change in an emergency, it should not be forgotten that the scientific principles that shape research methodology and safeguard scientific validity do not change. The published results should be treated accordingly.

Probability sampling is more expensive than convenience or quota sampling, and measurement using diagnostic interviews is more expensive than ad hoc items or screening scales. Nevertheless, the value proposition is greater. A single study incorporating these principles delivers valuable information in the form of valid estimates. The knowledge delivered by such studies brings more value by allowing linkage of decision-making to evidence. Dozens of potentially inaccurate estimates do not return the same value and have the potential to do harm.

It is not too late to at least partially address these deficiencies. Diagnostic instruments designed for use in surveys can assess period prevalence retrospectively. Furthermore, detailed studies using health administrative data will be possible once the data are made available to researchers and linked to other sources. Mental health policy, mental health care, and mental health–related interventions must be built on robust, valid data, efficiently collected, effectively managed, and properly interpreted. This pandemic has presented an opportunity to address this important issue so that the best data possible will be available to guide policy and investment in the future. A national initiative, perhaps led by Health Canada, with the collaboration of experts in the field of psychiatric epidemiology and administrative data along with, for example, Statistics Canada and the Canadian Institute for Health Information could use this opportunity to provide a better foundation for evidence in the future.

## References

[1] Mamelund SE . The impact of influenza on mental health in Norway, 1872-1929. Workshop, Carlseberg Academy; 2010. Accessed April 8, 2021. http://misms.net/wp-content/uploads/2016/10/CPH_abstracts.pdf

[2] Chen S She R Qin P , et al. The medium-term impact of covid-19 lockdown on referrals to secondary care mental health services: a controlled interrupted time series study. Front Psychiatry. 2020;11:585915. 33324258 10.3389/fpsyt.2020.585915 PMC7726266

[3] Kurdyak P . Brief for the standing committee on health: study: emergency situation facing Canadians in light of the second wave of the COVID-19 pandemic 2020. Accessed April 8, 2021. https://www.ourcommons.ca/DocumentViewer/en/43-2/HESA/meeting-11/evidence

[4] Taquet M Luciano S Geddes JR Harrison PJ . Bidirectional associations between COVID-19 and psychiatric disorder: retrospective cohort studies of 62 354 COVID-19 cases in the USA. Lancet Psychiatry. 2020;8(2):130–140. 33181098 10.1016/S2215-0366(20)30462-4 PMC7820108

[5] Winkler P Formanek T Mlada K , et al. Increase in prevalence of current mental disorders in the context of COVID-19: analysis of repeated nationwide cross-sectional surveys. Epidemiol Psychiatr Sci. 2020;29:e173. 32988427 10.1017/S2045796020000888 PMC7573458

[6] Pappa S Ntella V Giannakas T Giannakoulis VG Papoutsi E Katsaounou P . Prevalence of depression, anxiety, and insomnia among healthcare workers during the COVID-19 pandemic: a systematic review and meta-analysis. Brain Behav Immun. 2020;88:901–907. 32437915 10.1016/j.bbi.2020.05.026 PMC7206431

[7] Salari N Hosseinian-Far A Jalali R , et al. Prevalence of stress, anxiety, depression among the general population during the COVID-19 pandemic: a systematic review and meta-analysis. Global Health. 2020;16(1):57. 32631403 10.1186/s12992-020-00589-w PMC7338126

[8] McGinty EE Presskreischer R Han H Barry CL . Psychological Distress and Loneliness Reported by US Adults in 2018 and April 2020. JAMA. 2020;324(1):93–94. 32492088 10.1001/jama.2020.9740 PMC7270868

[9] Pan KY Kok AAL Eikelenboom M , et al. The mental health impact of the COVID-19 pandemic on people with and without depressive, anxiety, or obsessive-compulsive disorders: a longitudinal study of three Dutch case-control cohorts. Lancet Psychiatry. 2020;8(2):121–129. 33306975 10.1016/S2215-0366(20)30491-0 PMC7831806

